# Association Between Comorbidity Clusters and Mortality in Patients With Cancer: Predictive Modeling Using Machine Learning Approaches of Data From the United States and Hong Kong

**DOI:** 10.2196/71937

**Published:** 2025-07-16

**Authors:** Chun Sing Lam, Rong Hua, Herbert Ho-Fung Loong, Chun-Kit Ngan, Yin Ting Cheung

**Affiliations:** 1 School of Pharmacy, Faculty of Medicine, Chinese University of Hong Kong, 8th Floor, Lo Kwee-Seong Integrated Biomedical Sciences Building, Area 39, The Chinese University of Hong Kong, Shatin, N.T, Hong Kong, China, 852 39436833; 2 Department of Clinical Oncology, Faculty of Medicine, Chinese University of Hong Kong, Hong Kong, China; 3 Data Science Program, Worcester Polytechnic Institute, Worcester, United States; 4 Hong Kong Hub of Pediatric Excellence, Chinese University of Hong Kong, Hong Kong, China

**Keywords:** comorbidity, multimorbidity, machine learning, cluster, clustering, cancer, mortality, oncology, multiple chronic conditions, metabolic

## Abstract

**Background:**

Patients with cancer and cancer survivors often experience multiple chronic health conditions, which can impact symptom burden and treatment outcomes. Despite the high prevalence of multimorbidity, research on cancer prognosis has predominantly focused on cancers in isolation. There is growing interest in machine learning techniques for cancer studies. However, these methods have not been applied in the context of supportive care for patients with cancer who have multimorbidity. Furthermore, few studies have investigated the associations between comorbidity clusters and mortality outcomes.

**Objective:**

This study investigated comorbidity clusters among patients with cancer using machine learning and examined their associations with mortality outcomes in two large representative samples from the United States and Hong Kong.

**Methods:**

This study used data from the National Health and Nutrition Examination Survey (NHANES) and the Hospital Authority Data Collaboration Laboratory (HADCL). Participants aged ≥20 years with a history of cancer were included. The study used a two-step framework to identify clusters of comorbidities in NHANES. In the first step, we used four machine learning techniques, including the Bernoulli mixture model and partition-based methods, to cluster the comorbidities. In the second step, domain experts reviewed and ranked the identified clusters to ensure clinical relevance. The clusters that had the highest average rank were selected for further analysis. The associations between comorbidity clusters and mortality outcomes were analyzed using Cox proportional hazards models. We conducted an external validation to evaluate the generalizability of the clusters identified in the NHANES cohort and their associations with mortality using HADCL. The same number of clusters was replicated based on the distinctive patterns and distribution of comorbidities observed within each cluster.

**Results:**

The study included 4390 participants in NHANES and 12,484 participants in HADCL. Four comorbidity clusters were identified: low comorbidity, metabolic, cardiovascular disease (CVD), and respiratory. In NHANES, participants in the respiratory cluster had the highest risk of all-cause mortality (adjusted hazard ratio [aHR] 1.62, 95% CI 1.26‐2.08; *P<*.001), followed by the CVD cluster (aHR 1.50, 95% CI 1.26‐1.80; *P<*.001) compared to the low comorbidity cluster. The 3 clusters were associated with higher risks of CVD-related mortality (aHR 1.48‐3.05, 95% CI 1.14-4.07; *P*<.003). The effects of comorbidity clusters on mortality were modified by income-to-poverty ratio (*P* for interaction=.04), diet quality (*P* for interaction=.02), and cancer prognosis (*P* for interaction=.005). In the HADCL (validation) cohort, participants in the respiratory and CVD clusters had a higher risk of all-cause mortality.

**Conclusions:**

High comorbidity burden clusters showed increased all-cause and CVD-related mortality in patients with cancer. These findings highlight the significance of considering comorbidity burden in cancer care. Machine learning approaches can provide valuable insights into complex multimorbidity profiles. Further research is needed to deepen understanding of the relationships between multimorbidity and cancer-specific outcomes.

## Introduction

Advancements in cancer treatment have significantly increased survival rates and life expectancy for patients with cancer [1]. However, survivors may also experience multiple chronic health conditions. The prevalence of multimorbidity, which refers to the presence of two or more medical conditions simultaneously, is steadily rising with improvements in life expectancy [2,3]. In the United States, 40% of patients with cancer have at least one other chronic condition, and 15% have two or more comorbidities [4]. Comorbidities are believed to influence cancer detection, treatment uptake, and treatment toxicity [5,6]. Hence, there is an urgent need to shift the focus of health care from individual diseases to a more comprehensive approach that considers clusters of medical conditions [2]. Despite the high prevalence of multimorbidity, clinical and epidemiological research on cancer prognosis has largely focused on cancers in isolation. It is crucial to recognize that the co-occurrence of chronic health conditions can impact symptom burden and treatment outcomes in patients with cancer [7].

Recently, there has been growing interest in machine learning techniques, including unsupervised and supervised learning, for use in cancer detection, classification, staging, and treatment evaluation [8]. However, these methods have not been extensively applied in the context of supportive care for patients with cancer who have multimorbidity. Several studies have used clustering methods or factor analysis to identify clusters of chronic conditions or symptoms among patients with cancer [9-12], but few studies have investigated the associations between these clusters and survival outcomes. A rare example is the study of Hahn et al [13], who used latent class analysis to identify 4 comorbidity classes and found that clusters characterized by cardiovascular diseases (CVDs), diabetes, and chronic obstructive pulmonary disease were associated with worse overall survival rates in patients with colorectal cancer. It is currently unclear whether similar associations hold for people with other cancers.

There is a clear need to identify comorbidity clusters to provide prognostic information regarding cancer and coexisting health conditions [5]. Such information could greatly assist in making treatment decisions for patients with cancer who have comorbidities. Therefore, the objective of this predictive modeling study was to investigate clusters of comorbidities among patients with cancer in a nationally representative sample and examine their associations with survival outcomes. We also attempted to validate the association finding between comorbidity clustering patterns identified from machine learning and mortality using another large representative cohort from a different geographical location.

## Methods

The reporting of the study adheres to the Guidelines for Developing and Reporting Machine Learning Predictive Models in Biomedical Research (Table S1 in Multimedia Appendix 1) [14].

### Part 1: Cluster Identification (National Health and Nutrition Examination Survey Data)

#### Study Population

This retrospective study used data from 10 National Health and Nutrition Examination Survey (NHANES) survey cycles, a periodic cross-sectional survey conducted from 1999 to 2018 [15]. The NHANES assessed the health and nutritional status of a nationally representative sample of the civilian population in the United States. Detailed information about the sampling methodology has been reported elsewhere [15]. The NHANES was approved by the National Center for Health Statistics Institutional Review Board, and informed consent was obtained from all participants.

We included participants aged ≥20 years with a self-reported history of cancer in this study. They were asked, “Have you ever been told by a doctor or other health professional that you had cancer or a malignancy of any kind?” A positive response to this question indicated a cancer diagnosis. We excluded participants if they (1) were diagnosed solely with nonmelanoma skin cancer and had no other cancer types or (2) did not report the age at which they were diagnosed with cancer.

#### Covariates

Information on sociodemographic and lifestyle characteristics was collected through at-home interviews. This included the participants’ sex, age, ethnicity, education level, income level, smoking status, alcohol consumption, physical activity, diet, and supplement use (Table S2 in Multimedia Appendix 1). Body weight and height measurements were taken at a mobile examination center. The Healthy Eating Index (HEI) score, a validated measure of diet quality, was calculated using dietary recall data to evaluate conformance with federal dietary guidelines in the United States [16]. They also provided information about their age at cancer diagnosis and the type of cancer diagnosed. We further classified the cancer diagnoses based on their prognosis according to US statistics [17,18].

#### Ascertainment of Comorbidities

Fifteen specific conditions were consistently assessed in all waves of the survey. These conditions included CVDs (ie, congestive heart failure, coronary heart diseases, angina, heart attack [myocardial infarction], and stroke) [19], metabolic syndromes (hypertension, diabetes, and hyperlipidemia) [20], respiratory diseases (chronic bronchitis, asthma, and emphysema), arthritis, liver conditions, thyroid problems, and kidney disease. For most comorbidities, the participants were categorized as having a specific condition if they answered “Yes” to the question: “Has a doctor or other health professional ever told you that you have [condition]?” In addition to self-reported information, this study also defined diabetes (fasting glucose level or glycated hemoglobin A_1c_ level) and hypertension (systolic or diastolic blood pressure) based on quantitative measurements.

#### Clustering of Comorbidities

The study used a 2-step framework to identify clusters of comorbidities. In the first step, we used 4 machine learning techniques to cluster the comorbidities based on the binary (categorical) nature of the comorbidity data. One of these was the Bernoulli mixture model. Mixture models have previously been applied to clustering and dimensionality reduction problems [21,22]. The Bernoulli variant was chosen here, as previous studies have shown its suitability for modeling binary data [21,23]. The three other models were partition-based methods, which divide the data into a predefined number of partitions corresponding to the number of clusters [24]. Due to the highly sensitive nature of noises and outliers, traditional K-means algorithms are not suitable for clustering categorical data. Instead, K-modes and K-medoids should be used. K-modes replace means with modes to find the clusters and use a simple matching dissimilarity measure for clustering the data objects, which were characterized by the categorical attributes only [25], while K-medoids select an actual and representable data object for each cluster in each iteration that is the most centrally located object within the cluster [26]. The final approach was based on K-medoids and incorporated a bisecting methodology [27]. Previous studies have successfully applied these methods to identify clusters of symptoms [28], clinical prognostic features in oncology [29], and comorbidities with other chronic diseases [30].

In the second step, the study incorporated domain knowledge into the interpretation of the clusters [31,32]. As this step required knowledge in clinical oncology, survivorship, and data analytics, domain experts who were clinicians or clinician researchers with relevant experience were invited to review the results from the first step. One medical oncologist (HHL), one cancer epidemiologist (YTC), one pharmacist (CSL), and one data scientist (CN) participated in this step. First, they were provided with the clustering results from the four approaches without identification of the specific method used. Then, they were asked to examine the relative distribution of comorbidities across the clusters generated by the machine learning methods and to assess the distinguishability of patterns of comorbidities between the clusters. After that, they ranked the most clinically relevant clusters. The clusters with the highest average rank were selected for further analysis to investigate their associations with mortality outcomes. We also used performance metrics, including Silhouette analyses, Calinski-Harabasz index, and Davies-Bouldin index, to support the selection [33,34]. This ensured that the clusters chosen for detailed investigation were those with the most potential to provide clinical insights into the relationship between comorbidities and mortality in patients with cancer. The clustering process was performed using Python (version 3.10; Python Software Foundation) and R (version 4.0.1; R Foundation).

#### Mortality Outcomes

The NHANES was linked to the death certificate records from the National Death Index using a probabilistic match method [35]. The participants were followed up from the date of interview to the date of death or December 31, 2019, whichever came first (the last date for available mortality data). Causes of death were coded using the *ICD-10* (*International Statistical Classification of Diseases, 10th Revision*). The primary outcomes comprised all-cause mortality and the top three cause-specific mortalities: cancer (*ICD-10* codes C00-C97), CVD (*ICD-10* codes I00-I09, I11, I13, I20-I51, and I60-I69), and respiratory diseases (*ICD-10* codes J40-J47).

#### Statistical Analysis

We conducted data analyses in accordance with the NHANES guidelines [15]. The survey design was taken into account by applying sample weights, clustering, and stratification in all analyses. Participants with missing data on death, comorbidities, or other covariates were excluded from the study.

Cox proportional hazards models were used to examine the associations between comorbidity clusters and mortality outcomes. We ran three models: model 1 (unadjusted), model 2 (adjusted for the age and sex of the participants), and model 3 (further adjusted for socioeconomic factors [educational level, ethnicity, and income-to-poverty ratio], lifestyle behaviors [BMI, HEI score, smoking status, alcohol drinking, physical activity, and supplement use], cancer prognosis, and time since the cancer diagnosis). Multiple imputation using the MICE (Multivariate Imputation by Chained Equations) package was conducted to address missing values [36]. We conducted stratified analyses to assess potential effect modification by covariates on the associations between comorbidity clusters and mortality.

Statistical analyses were carried out using SAS 9.4 (SAS Institute Inc) and R 4.0.1 (R Foundation). A *P*<.05 was considered statistically significant.

### Part 2: Cluster Verification (Hospital Authority Data Collaboration Laboratory Data)

#### Study Population

In Hong Kong, the Hospital Authority is a statutory body that governs all public hospital services. These hospitals provide approximately 90% of all secondary and tertiary care services in Hong Kong [37]. Hospital Authority Data Collaboration Laboratory (HADCL) provides comprehensive deidentified data, including sociodemographic details, clinical diagnoses, medications, and hospital admission data from the Hospital Authority, and has been used in large-scale epidemiological studies [38]. A large subset of the data, including approximately 200,000 participants who used public health care services in 2007 and 2017, was accessed through a self-service data platform. To ensure the representativeness of the sample, HADCL used a proportionate random sampling approach [39]. This HADCL cohort is a relatively representative sample of the general population in Hong Kong and has been used widely in epidemiological studies [40,41].

For this study, we included participants diagnosed with malignant cancer (*ICD-10* code C00-C97). Similar to the NHANES cohort, we excluded participants if they were (1)<20 years of age, (2) diagnosed solely with nonmelanoma skin cancer (*ICD-10* code C44) and had no other cancer types.

#### Covariates

Information on sociodemographic and clinical characteristics was collected from the data repository. They included the individuals’ sex, residential area, age at cancer diagnosis, and cancer site. The income level of individuals was determined based on the income level of their residential areas (Table S2 in Multimedia Appendix 1). Cancer diagnoses were further classified according to their prognosis, using classifications consistent with our previous NHANES cohort and local statistics [42].

#### Ascertainment and Clustering of Comorbidities

The HADCL data contained clinical diagnoses of patients documented using *ICD-10* codes. Based on the NHANES cohort, patient diagnoses of the same fifteen comorbidities (congestive heart failure, coronary heart diseases, angina, heart attack [myocardial infarction], stroke, hypertension, diabetes, hyperlipidemia, chronic bronchitis, asthma, emphysema, arthritis, liver conditions, thyroid problems, and kidney disease) were collected.

This part is an external validation study to evaluate the generalizability of the comorbidity clusters identified in the NHANES cohort and their associations with mortality within a different group of patients [43]. As this was an external validation, instead of a de novo clustering analysis, we replicated the same number of clusters based on the distinctive patterns and distribution of comorbidities observed within each cluster identified in the main cohort [44,45]. We then examined whether the associations between these clusters and all-cause mortality remained consistent.

#### Mortality Outcomes and Statistical Analysis

The date of death was documented in the HADCL dataset. Participants were followed up until the date of death or the end of follow-up (ie, June 1, 2021, which was the last day of captured data available in the database), whichever occurred first. The index date of the study was defined as the date when individuals first received a diagnosis of malignant cancer. Cox proportional hazards models were used to examine the associations between comorbidity clusters and all-cause mortality. Similarly, three models were used: the crude model (model 1), model adjusted for age at cancer diagnosis and sex (model 2), and model adjusted for age at cancer diagnosis, sex, income level of residential district, and cancer prognosis (model 3). Participants with missing data on death, comorbidities, or other covariates were excluded from the study.

Statistical analyses were carried out using R (version 4.0.1; R Foundation). A *P*<.05 was considered statistically significant.

### Ethical Considerations

This study was approved by the Survey and Behavioural Research Ethics Committee of the Chinese University of Hong Kong (SBRE-23‐0014), which allowed secondary analysis of HADCL and NHANES data without additional consent. The NHANES study protocol was approved by the Research Ethics Review Board of the National Center for Health Statistics, and informed consent was obtained from all NHANES participants. The data from HADCL and NHANES were deidentified.

## Results

### Characteristics of Participants in the NHANES Cohort

**Figure 1.**
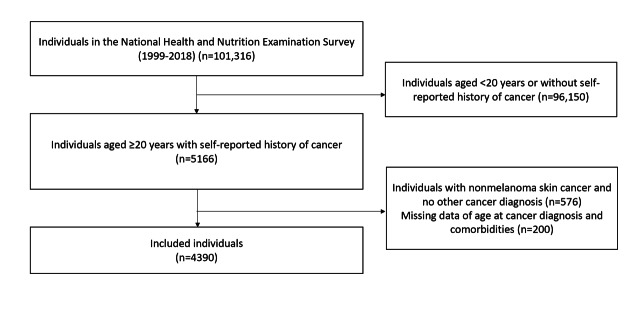
Flowchart of individual inclusion and exclusion in the National Health and Nutrition Examination Survey cohort.

From the total of 101,316 individuals in the 1999‐2018 waves of the NHANES, those without a diagnosis of cancer (n=96,150), those with a sole diagnosis of nonmelanoma skin cancer (n=576), and those with missing data (n=200) were excluded. Ultimately, the analysis included 4390 individuals (Figure 1).

Table 1 presents the characteristics of the included individuals. The mean age of the participants was 66 (SD 14.6) years, and 54% were female (n=2376). The majority of them were non-Hispanic White (n=2870, 65.4%). The median time since cancer diagnosis was 7 (IQR 3-15) years, and the mean age at cancer diagnosis was 55.4 (SD 17.6) years. Among the individuals, the most common cancer diagnoses were genitourinary cancer (n=1102, 25.1%). More than half of them self-reported a diagnosis of hypertension (n=2812, 64.1%), while approximately half reported having hyperlipidemia (n=2234, 50.9%) and approximately half reported having arthritis (n=2206, 50.3%).

**Table 1.** Summary of characteristics of individuals diagnosed with cancer in the National Health and Nutrition Examination Survey cohort (N=4390)

Characteristic	Value
Sociodemographic	
Sex, n (%)	
Male	2014 (45.9)
Female	2376 (54.1)
Age (years), mean (SD)	66.0 (14.6)
Education level, n (%)	
Below college	2154 (49.1)
College or above	2236 (50.9)
Family income to poverty, mean (SD)	2.65 (1.59)
≤1.3	1184 (27.0)
1.3‐3.5	1803 (41.0)
>3.5	1404 (32.0)
Ethnicities, n (%)	
Mexican American	344 (7.8)
Non-Hispanic White	2870 (65.4)
Non-Hispanic Black	703 (16.0)
Others	473 (10.8)
Clinical	
Age at cancer diagnosis, mean (SD)	55.4 (17.6)
Time since cancer diagnosis, median (IQR)	7 (3-15)
Type of cancer, n (%)	
Breast cancer	815 (18.6)
Digestive or gastrointestinal cancer	536 (12.2)
Genitourinary cancer	1102 (25.1)
Gynecological cancer	694 (15.8)
Skin cancer	762 (17.4)
Head and neck cancer	173 (3.9)
Respiratory or thoracic cancer	152 (3.5)
Others	552 (12.6)
Cancer prognosis^**a**^, n (%)	
Highest	2512 (57.2)
Middle	1435 (32.7)
Lowest	443 (10.1)
Comorbidities, n (%)	
Hyperlipidemia	2234 (50.9)
Hypertension	2812 (64.1)
Arthritis	2206 (50.3)
Heart failure	347 (7.9)
Coronary heart diseases	426 (9.7)
Angina	289 (6.6)
Heart attack	443 (10.1)
Stroke	404 (9.2)
Bronchitis	452 (10.3)
Liver condition	255 (5.8)
Kidney diseases	338 (7.7)
Diabetes	1074 (24.5)
Asthma	678 (15.4)
Thyroid diseases	320 (7.3)
Emphysema	263 (6.0)
Lifestyle	
BMI, mean (SD)	28.9 (6.6)
<25 kg/m^2^ (normal)	1266 (28.8)
25‐30 kg/m^2^ (overweight)	1549 (35.3)
≥30 kg/m^2^ (obese)	1575 (35.9)
Smoking status, n (%)	
Never smokers	1923 (43.8)
Former smokers	1766 (40.2)
Current smokers	701 (16.0)
Drinking status, n (%)	
Nondrinker	2202 (50.2)
Low-to-moderate drinker	1885 (42.9)
Heavy drinker	303 (6.9)
Physical activity, n (%)	
Physically active (≥150 h/wk)	1158 (26.4)
Irregularly active (<150 h/wk)	680 (15.5)
Inactive	2552 (58.1)
Healthy Eating Index, median (IQR)	52 (42-63)
<51.55	2194 (50.0)
≥51.55	2196 (50.0)
Supplement use (≥90 days), n (%)	2646 (60.3)

a Cancer prognosis was based on the US statistics: group 1 (highest: average 5-year survival rate ≥90%), group 2 (middle: average 5-year survival rate ≥60% and <90%), and group 3 (lowest: average 5-year survival rate <60%).

### Characteristics of Participants in the HADCL Cohort

**Figure 2.**
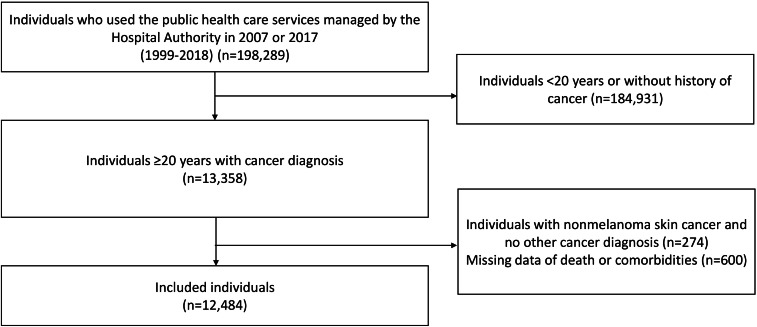
Flowchart of individual inclusion and exclusion in the Hospital Authority Data Collaboration Laboratory cohort.

From the total of 198,289 individuals in the HADCL cohort, those without a diagnosis of cancer or younger than 20 years (n=184,931), those with a sole diagnosis of nonmelanoma skin cancer (n=274), and those with missing data (n=600) were excluded. Ultimately, 12,484 individuals were included in the analysis (Figure 2).

Table 2 presents the characteristics of the included individuals. Their mean age was 60.9 (SD 14.4) years, and 52.7% were female (n=6584). The mean age at cancer diagnosis was 64.8 years. The most common cancer diagnoses were digestive cancer (n=3938, 31.5%). The most common comorbidity was hypertension (n=3464, 27.7%), followed by diabetes (n=2053, 16.4%).

**Table 2.** Summary of characteristics of individuals diagnosed with cancer in the Hospital Authority Data Collaboration Laboratory cohort (N=12,484).

Characteristic	Value
Sociodemographic	
Sex, n (%)	
Male	5900 (47.3)
Female	6584 (52.7)
Age (years), mean (SD)^a^	60.9 (14.4)
Income level^b^, n (%)	
Lowest-income	5332 (42.7)
Middle-income	3642 (29.2)
Highest-income	3510 (28.1)
Clinical, mean (SD)	
Age at cancer diagnosis	64.8 (14.7)
Type of cancer, n (%)	
Cancers of lip, oral cavity, and pharynx (C00-C14)	863 (6.9)
Cancers of digestive organs (C15-C26)	3938 (31.5)
Cancers of respiratory and intrathoracic organs (C30-C39)	1741 (13.9)
Cancers of bone and articular cartilage (C40-C41)	50 (0.4)
Malignant melanoma of skin (C43)	49 (0.4)
Cancers of mesothelial and soft tissue (C45-C49)	162 (1.3)
Breast cancer (C50)	2242 (18.0)
Cancers of female genital organs (C51-C58)	998 (8.0)
Cancers of male genital organs (C60-C63)	898 (7.2)
Cancers of the urinary tract (C64-C68)	772 (6.2)
Cancer of the eye, brain, and other parts of the CNS (C69-C72)	96 (0.8)
Cancers of the thyroid and other endocrine glands (C73-C75)	430 (3.4)
Cancers of ill-defined, secondary, and unspecified sites (C76-C80)	3172 (25.4)
Cancers of primary, lymphoid, hematopoietic, and related tissue (C81-C96)	810 (6.5)
Cancer prognosis^c^, n (%)	
Highest	2819 (22.6)
Middle	3839 (30.7)
Lowest	5826 (46.7)
Comorbidities, n (%)	
Hyperlipidemia	1136 (9.1)
Hypertension	3464 (27.7)
Arthritis	513 (4.1)
Heart failure	801 (6.4)
Coronary heart disease	1047 (8.4)
Angina	397 (3.2)
Heart attack	484 (3.9)
Stroke	905 (7.2)
Bronchitis	137 (1.1)
Liver condition	1265 (10.1)
Kidney diseases	712 (5.7)
Diabetes	2053 (16.4)
Asthma	246 (2.0)
Thyroid diseases	623 (5.0)
Emphysema	14 (0.1)

a This refers to the age in 2007 (the first time point for patient sampling).

b The income level is based on the residential areas of individuals, categorized into 3 groups based on median monthly household income.

c Cancer diagnoses were further classified according to their prognosis, based on previous classification in the National Health and Nutrition Examination Survey cohort and local statistics.

### Clustering of Comorbidities Using the NHANES Cohort

Figure S1 in Multimedia Appendix 1 shows the clusters generated using the 4 selected approaches. According to the reviews of domain experts, the Bernoulli mixture model ranked the highest (unanimously ranked highest by all experts based on the distinguishability of clusters and clinical relevance), followed by bisecting K-medoids and K-medoids, while K-modes ranked the lowest due to its inability to identify distinguishable clusters. Table S3 in Multimedia Appendix 1 presents the evaluation of clustering results from the 4 approaches using Silhouette analyses, Calinski-Harabasz index, and Davies-Bouldin index. It also shows the quality of the clusters generated by the Bernoulli mixture model being the highest among the 4 approaches.

Based on the distribution patterns of comorbidities in the NHANES cohort, 4 clusters derived from the Bernoulli mixture model were chosen due to the notable differences in comorbidity patterns across the clusters. Table S4 in Multimedia Appendix 1 presents the characteristics of the individuals in each of the 4 clusters, which comprised 2127 (48.5%), 1525 (34.7%), 421 (9.6%), and 317 (7.2%) individuals.

**Figure 3.**
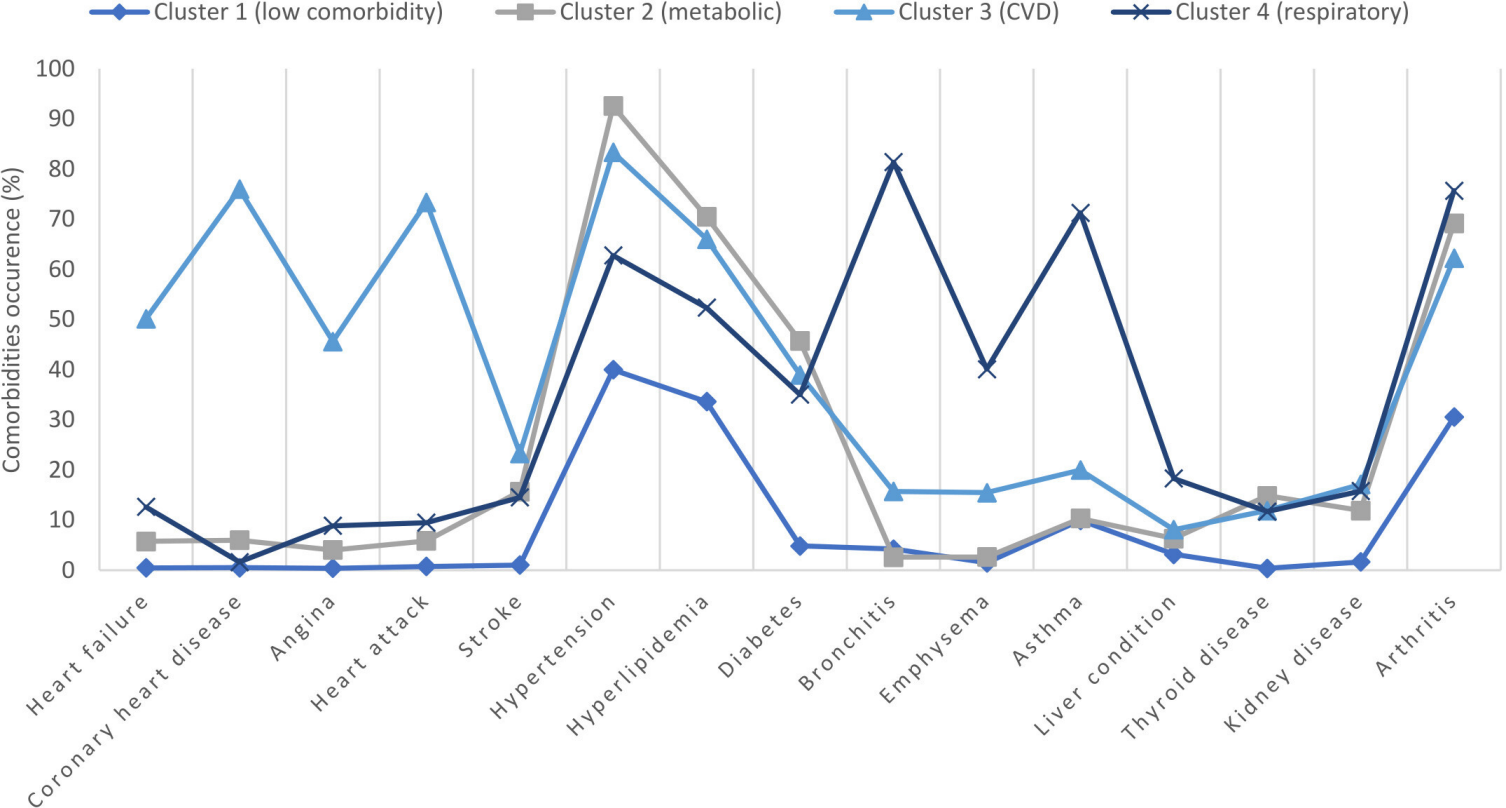
Distribution of comorbidities among the 4 comorbidity clusters in the National Health and Nutrition Examination Survey cohort. CVD: cardiovascular disease.

Figure 3 and Table S5 in Multimedia Appendix 1 illustrate the comorbidity patterns observed in the 4 clusters. The patients in cluster 1 (low comorbidity cluster) exhibited significantly lower percentages of all comorbidities than the patients in the other clusters. Cluster 2 (metabolic cluster) was characterized by the highest burden of metabolic syndrome (hypertension, hyperlipidemia, and diabetes) among all 4 clusters. Cluster 3 (CVD cluster) was characterized by the highest burden of CVD among the clusters and also displayed a relatively high burden of metabolic syndromes (although lower than that of cluster 2). Cluster 4 (respiratory cluster) exhibited a significantly higher burden of respiratory diseases, while also having a moderate burden of metabolic syndromes (although lower than those of clusters 2 and 3).

### Clustering of Comorbidities Using the HADCL Cohort

Based on the comorbidity clusters identified in the NHANES cohort, 4 clusters were manually categorized according to observed patterns and the distribution of comorbidities within those clusters. Individuals with metabolic diseases were first grouped into cluster 2 (metabolic cluster), and then those with CVD or respiratory comorbidities were assigned to cluster 3 (CVD cluster) and cluster 4 (respiratory cluster), respectively. The remaining individuals were classified into cluster 1 (low comorbidity). Table S6 in Multimedia Appendix 1 shows the characteristics of the individuals in the 4 clusters, which comprised 7392 (59.2%), 2521 (20.2%), 2188 (17.5%), and 383 (3.1%) participants. Figure S2 and Table S7 in Multimedia Appendix 1 illustrate the comorbidity patterns observed in the 4 clusters.

### Association Between Comorbidity Clusters and Mortality Outcomes in the NHANES Cohort

After a median follow-up of 6.6 (IQR 3.3‐11) years, 1700 deaths (38.7% of individuals) were recorded. As shown in Table 3, all 3 models suggested that there was a significant association between the comorbidity clusters and all-cause mortality. After adjusting for confounders (model 3), compared with the low comorbidity cluster, the individuals in the respiratory cluster had the highest risk of mortality (adjusted hazard ratio [aHR] 1.62, 95% CI 1.26‐2.08; *P<*.001), followed by the CVD cluster (aHR 1.50, 95% CI 1.26‐1.80; *P<*.001) and the metabolic cluster (aHR 1.15, 95% CI 1.02‐1.29; *P=*.03).

**Table 3.** Associations of comorbidity clusters with all-cause and cause-specific mortality in the National Health and Nutrition Examination Survey and Hospital Authority Data Collaboration Laboratory cohort.

Cohort and cluster	Number of patients, n (%)	Death, n (%)	Model 1, hazard ratio (95% CI)	*P* value	Model 2^a^, hazard ratio (95% CI)	*P* value	Model 3^a^, hazard ratio (95% CI)	*P* value
NHANES^b^ cohort								
All-cause mortality								
Cluster 1 (low comorbidity)	2127 (48.5)	657(30.9)	Ref^c^	—^d^	Ref	—	Ref	—
Cluster 2 (metabolic)	1525 (34.7)	650 (42.7)	2.10 (1.84‐2.39)	<.001	1.24 (1.10‐1.39)	<.001	1.15 (1.02‐1.29)	.03
Cluster 3 (CVD^e^)	421 (9.6)	261 (62.0)	3.75 (3.03‐4.64)	<.001	1.78 (1.49‐2.14)	<.001	1.50 (1.26‐1.80)	<.001
Cluster 4 (respiratory)	317 (7.2)	132 (41.6)	1.97 (1.52‐2.54)	<.001	1.92 (1.51‐2.42)	<.001	1.62 (1.26‐2.08)	<.001
Cancer mortality (C00-C97)								
Cluster 1 (low comorbidity)	2127 (48.5)	253 (11.9)	Ref	—	Ref	—	Ref	—
Cluster 2 (metabolic)	1525 (34.7)	213 (14.0)	1.48 (1.19‐1.84)	<.001	1.00 (0.80‐1.25)	.98	0.92 (0.73‐1.17)	.50
Cluster 3 (CVD)	421 (9.6)	61 (14.5)	1.92 (1.28‐2.88)	.002	1.07 (0.71‐1.60)	.75	0.89 (0.59‐1.33)	.57
Cluster 4 (respiratory)	317 (7.2)	48 (15.1)	1.46 (0.94‐2.25)	.09	1.50 (0.96‐2.33)	.07	1.20 (0.77‐1.87)	.43
CVD mortality (I00-I09, I11, I13, I20-I51, and I60-69)								
Cluster 1 (low comorbidity)	2127 (48.5)	130 (6.1)	Ref	—	Ref	—	Ref	—
Cluster 2 (metabolic)	1525 (34.7)	174 (11.4)	2.99 (2.24‐3.99)	<.001	1.57 (1.21‐2.05)	<.001	1.48 (1.14‐1.93)	.003
Cluster 3 (CVD)	421 (9.6)	101 (24.0)	8.45 (6.20‐11.5)	<.001	3.54 (2.68‐4.69)	<.001	3.05 (2.29‐4.07)	<.001
Cluster 4 (respiratory)	317 (7.2)	32 (10.1)	2.58 (1.64‐4.08)	<.001	3.13 (1.56‐3.86)	<.001	2.19 (1.35‐3.54)	.001
Respiratory mortality (J40-J47)								
Cluster 1 (low comorbidity)	2127 (48.5)	29 (1.4)	Ref	—	Ref	—	Ref	—
Cluster 2 (metabolic)	1525 (34.7)	26 (1.7)	2.79 (1.45‐5.38)	.002	1.53 (0.75‐3.12)	.24	1.29 (0.61‐2.72)	.51
Cluster 3 (CVD)	421 (9.6)	18 (4.3)	7.39 (3.81‐14.3)	<.001	3.38 (1.64‐6.98)	<.001	2.09 (0.97‐4.47)	.06
Cluster 4 (respiratory)	317 (7.2)	18 (5.7)	6.51 (3.29‐12.9)	<.001	5.94 (2.99‐11.8)	<.001	3.99 (2.03‐7.83)	<.001
HADCL^f^ cohort								
All-cause mortality								
Cluster 1 (low comorbidity)	7392 (59.2)	1747(23.6)	Ref	—	Ref	—	Ref	—
Cluster 2 (metabolic)	2521 (20.2)	905 (35.9)	1.65 (1.52‐1.79)	<.001	1.11 (1.02‐1.21)	.01	1.08 (0.99‐1.17)	.08
Cluster 3 (CVD)	2188 (17.5)	1086 (49.6)	2.37 (2.19‐2.55)	<.001	1.78 (1.26‐1.48)	<.001	1.33 (1.23‐1.45)	<.001
Cluster 4 (respiratory)	383 (3.1)	184 (48.0)	2.22 (1.91‐2.58)	<.001	1.32 (1.13‐1.54)	<.001	1.32 (1.13‐1.54)	<.001

a In the NHANES cohort, model 2 was adjusted for age and sex, model 3 was adjusted for age at assessment, sex, socioeconomic status (education level, ethnicities, and income-to-poverty ratio), lifestyle behaviors (BMI, Healthy Eating Index, smoking and alcohol status, physical activity, and supplement use), years since cancer diagnosis, and cancer prognosis. In the HADCL cohort, model 2 was adjusted for age at cancer diagnosis and sex, and model 3 was adjusted for age at cancer diagnosis, sex, income level (based on the median household income of the districts), and cancer prognosis.

b NHANES: National Health and Nutrition Examination Survey.

c Ref: reference group.

d Not applicable.

e CVD: cardiovascular disease.

f HADCL: Hospital Authority Data Collaboration Laboratory.

Regarding cause-specific mortality, the metabolic, CVD, and respiratory clusters were associated with higher risks of CVD-related mortality than the low comorbidity cluster, with the CVD cluster (aHR 3.05, 95% CI 2.29‐4.07; *P<*.001) showing the highest risk. Only individuals in the respiratory cluster had a higher risk of respiratory disease mortality than those in the low comorbidity cluster (aHR 3.99, 95% CI 2.03‐7.83; *P<*.001) after adjusting for all confounders, although the metabolic and CVD clusters were also significantly associated with higher respiratory disease mortality in the crude models. However, no significant differences in the risk of cancer mortality were observed among the clusters (all *P>*.05).

**Figure 4.**
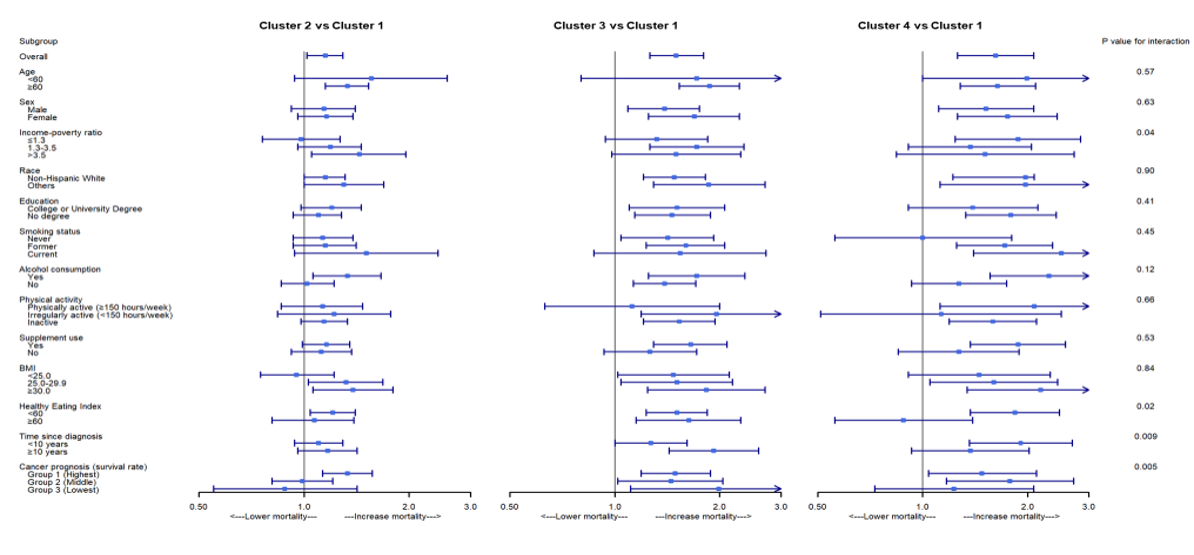
Effect modification by factors on the association of clusters with mortality in the National Health and Nutrition Examination Survey cohort (a more detailed figure is presented in Figure S4 in Multimedia Appendix 1).

The subgroup analysis (Figure 4) indicated that the effects of the comorbidity clusters on mortality were modified by the income-to-poverty ratio (*P* for interaction=.04), HEI score (*P* for interaction=.02), time since cancer diagnosis (*P* for interaction=.009), and cancer prognosis (*P* for interaction=.005) after adjusting for all confounders. The individuals in the respiratory cluster had a significantly higher risk of mortality (*P*=.003) if they had a lower income-to-poverty ratio, while those in the metabolic cluster (*P*=.02) had a higher risk of mortality if they reported a higher income-to-poverty ratio. Individuals with low HEI scores in all 3 other clusters had a higher risk of mortality than those with low HEI scores in the low comorbidity cluster (all *P*<.01). However, for individuals with high HEI scores, only those in the CVD cluster had a higher risk of mortality than those with high HEI scores in the low comorbidity cluster (*P*=.006).

### Verification of the Association Between Comorbidity Clusters and All-Cause Mortality in the HADCL Cohort

After a median follow-up of 8.9 (IQR 3.8‐13.5) years, 3922 deaths (31.4% of individuals) were recorded. As shown in Table 3, the crude model and the age- and sex-adjusted model suggested that there was a significant association between the comorbidity clusters and all-cause mortality. After adjusting for confounders (model 3), compared with the low comorbidity cluster, the individuals in the CVD cluster (aHR 1.33, 95% CI 1.23‐1.45; *P*<.001) and respiratory cluster (aHR 1.32, 95% CI 1.13‐1.54; *P*<.001) had a higher risk of mortality. The metabolic cluster also tends to have a higher risk of mortality compared with the low comorbidity cluster; however, the association was not significant (aHR 1.08, 95% CI 0.99‐1.17; *P*=.08).

## Discussion

### Principal Findings

This is one of the largest studies to use machine learning in identifying multimorbidity clusters and as a prognosis marker of mortality in patients with different types of cancer. This study focused on clustering comorbidities in 2 distinct patient cohorts from different geographical locations. The following four clusters were identified: low comorbidity (cluster 1), metabolic (cluster 2), cardiovascular (cluster 3), and respiratory (cluster 4) clusters. Compared with the low comorbidity cluster, the CVD and respiratory clusters were consistently associated with higher all-cause mortality in both cohorts, and all clusters were associated with higher CVD mortality rates in the NHANES cohort. No associations were identified between specific clusters of comorbidities and cancer mortality. These observed associations may inform the development of cluster-specific care management and clinical guidelines for common comorbidities for people living with and beyond cancer.

### Machine Learning Approaches in the Study of Multimorbidity

The use of machine learning is especially relevant in the study of comorbidities due to the complex interactions between cancer and other coexisting health conditions. Compared with traditional methods, unsupervised learning is more effective and flexible at identifying unknown patterns among patient subgroups, without the need for prior human knowledge and intervention (eg, determining cutoff values). In this study, mixture models outperformed the partition-based methods in distinguishing patterns across the identified clusters, which may be due to their advantages in clustering data with different shapes and sizes [46,47]. Also, Bernoulli mixture models are specifically designed for binary data, where each feature is a Bernoulli distribution [23]. In contrast, methods such as K-modes, K-medoids, and bisecting K-medoids are more general and suitable for categorical data but may not capture the nuances of binary data as Bernoulli mixture models do. Mixture models also offer other advantages to K-modes, K-medoids, and bisecting K-medoids; for instance, they can provide soft clustering assignments to allow for flexibility and handle missing data more effectively [48,49].

Consistent with emerging evidence supporting the role of artificial intelligence in health care [50], our findings suggest that, in the near future, such algorithms may be incorporated into health care systems as risk stratification tools to assist clinicians in identifying patients at risk of adverse outcomes. Clustering and other machine learning approaches have been used in the development of risk prediction models for various diseases. Notably, several of these models have included comorbidities as one of the components, such as in predicting the risk of complications in patients with diabetes [51] and mortality risk in patients with chronic obstructive pulmonary disease [52]. Hence, the clustering approach used in this study may be applied to assist in predicting mortality risk upon the initial cancer diagnosis.

### Comorbidity Clusters in Cancer

There are similarities between the comorbidity clusters identified in previous studies [13,53,54] and those in this study, despite some variations in the scopes of health conditions. For instance, clusters associated with cardiovascular and respiratory conditions have been observed in patients with breast, colorectal, and lung cancers [13,53,54]. This suggests that the multimorbidity profile may be similar across different types of cancer. Our study revealed that the increased risk of mortality across all three comorbidity clusters (compared with the low comorbidity cluster) remained in cancers with good prognoses. This finding is reasonable, as patients with cancers of poor prognosis are more likely to die from their cancer regardless of their comorbidity status [55]. Overall, the individuals in the respiratory and CVD clusters had a higher risk of all-cause mortality than individuals from other clusters. Recent data from the United States indicate that the majority of patients with cancer die from noncancer-related causes, most commonly heart disease [56,57]. In comparison, respiratory diseases account for a smaller proportion of noncancer-related deaths [56]. The fact that the patients in the respiratory cluster also experienced poor prognosis in our study suggests the need for improved screening and management of comorbidities in this group of patients. Many cancer treatment modalities, including radiotherapy, chemotherapy, and immunotherapy, can potentially lead to pulmonary toxicities [58-60]. Future epidemiological studies should explore how cancer and its treatment impact comorbidity outcomes, particularly regarding pulmonary conditions and their subsequent effects on survival outcomes. Conversely, this study did not find significant differences in cancer mortality across the four comorbidity clusters. One possible reason may be competing risks from mortality due to other causes, such as mortality from CVD. The high cardiovascular mortality risk in patients with a high comorbidity burden could overshadow any differences in cancer mortality that might be observed in later stages of life. Given the improving management and tighter control of cardiovascular risk factors in cancer patients over the recent years, it may be worthwhile to examine these associations again in future waves of data collection within NHANES.

Notably, our study uncovered a unique cluster that was prevalent among patients with cancer in the NHANES and HADCL cohorts. This cluster was characterized by metabolic syndromes, which are known risk factors for CVDs [20,61], but a low burden of CVD. Previous research has indicated that the prevalence of metabolic syndromes may be higher in patients with cancer or survivors of cancer than in individuals without cancer [61]. Cancer and metabolic syndromes share common risk factors, such as age, obesity, and lifestyle factors [62]. In terms of mortality outcomes, the CVD cluster exhibited an all-cause mortality rate 1.3 times higher and a CVD mortality rate more than 2 times higher than the rates observed for the metabolic cluster in the NHANES cohort. Considering the important connections between metabolic syndromes and CVD, patients in this cluster are likely to develop CVD if these risk factors are not well controlled. This finding underscores the importance of early interventions to manage metabolic risk factors and reduce CVD risk in patients with cancer.

### The Modification Effect of Lifestyle and Socioeconomic Factors

We observed effect modification of some lifestyle factors in the NHANES cohort. One important modifiable factor that may impact the associations between comorbidity clusters and mortality is diet. Our findings suggest that patients with multimorbidity who follow an unhealthy diet may have higher all-cause mortality rates in all 3 clusters compared with the low comorbidity cluster, with a particularly significant difference in the respiratory cluster. Previous studies have demonstrated an inverse relationship between adherence to healthy dietary patterns and mortality in patients with cancer and survivors of cancer [63,64]. In addition, diet quality has been found to be associated with multimorbidity. For instance, a Western dietary pattern is associated with an increased risk of multimorbidity [65]. Notably, metabolic syndrome, which was prevalent across the high comorbidity clusters, may serve as a surrogate marker for dietary risk factors in cancer [62]. Taken together, adopting a healthier diet may help reduce mortality in patients with cancer by lowering the incidence of chronic health conditions. Therefore, it is crucial to include dietary interventions as part of a holistic care plan for patients with cancer, especially those with high comorbidity burdens.

Another effect modifier to consider is the income-to-poverty ratio, which has been found to modify the association between comorbidity clusters and mortality in varying ways. Previous studies have shown that a lower socioeconomic status is associated with higher mortality rates in patients with cancer [66,67]. While it is known that comorbidities can contribute to inequities in survival, there is a lack of research exploring the impact of different comorbidities on the socioeconomic disparities in survival. Our study shows that a low socioeconomic status is associated with poorer survival in patients with cancer who have respiratory conditions, but the opposite is observed for patients with metabolic diseases. It has previously been demonstrated that modifiable risk factors, such as obesity and smoking, are important mediators of the associations between socioeconomic status and mortality [68,69]. Therefore, one possible explanation for the differences in the associations between socioeconomic status and mortality across comorbidity clusters may be variations in the mediating effects of these lifestyle factors. For example, mortality in patients with cancer who have respiratory comorbidities and a lower socioeconomic status may be more strongly influenced by smoking status and poor access to health care services, whereas the mortality risk in patients with metabolic diseases may be increased by other lifestyle factors associated with high socioeconomic status, such as a sedentary occupation, unhealthy dietary patterns, and excess alcohol consumption. Future studies should investigate the mediating effects of modifiable risk factors on the associations between socioeconomic status and mortality among patients with different comorbidities. This may assist the development of targeted interventions to reduce the inequities in mortality among patients with cancer.

### Strengths and Limitations

The strength of this study lies in its use of a nationally representative sample of patients with cancer to identify the clusters and the validation of the results using another large sample of patients from a different geographical location. However, there are several limitations to this study. First, the data for cancer and comorbidities were self-reported in the NHANES cohort, which may have introduced the possibility of recall bias. However, previous studies have shown generally good agreement between health records and self-reports for conditions including diabetes, hypertension, and myocardial infarction [70,71]. We further validated the associations between comorbidity clusters and mortality using the HADCL cohort, which includes documented disease diagnoses. Second, the study lacked information regarding the staging of cancer diagnosis. However, studies have shown that differences in survival at diagnosis between stage groups largely disappeared after having survived for 5-10 years [72]. As the main cohort in this study consists of survivors (with a median survival of 7 y) rather than patients on active treatment, the comorbidity clusters identified in this study may be more relevant to cancer survivors instead of newly diagnosed patients. Moreover, the study did not provide information on the severity and treatment of comorbidities. Previous studies have demonstrated that the impact of comorbidities increases with their severity, which may be due to differential effects on treatment toxicities and tolerance, direct impacts on cancer progression, or other factors [5]. These may limit the mechanistic insights into mortality drivers. Future studies should use electronic health records to ascertain these clinical characteristics for verification of the findings. The manually assigned clusters in the validation cohort (ie, the HADCL data) may introduce a certain degree of confirmation bias. However, the purpose of the validation cohort is to confirm the association between comorbidity clusters identified from the NHANES data and mortality; hence, we reckoned that a manual assignment approach is still reasonable. Future studies should use another external cohort to validate the identified clusters in our study.

### Conclusions

This study used machine learning techniques to investigate clusters of comorbidities and mortality outcomes among two large samples of patients with cancer in the United States and Hong Kong. Compared with individuals with low comorbidity burdens, those in the respiratory and CVD clusters showed higher all-cause mortality in both samples, and all 3 clusters showed higher CVD-related mortality rates in the NHANES cohort. However, no significant associations between these clusters and cancer-specific mortality were observed. Diet quality and socioeconomic status are effect modifiers of the associations between comorbidity clusters and mortality. Overall, the results demonstrate the potential of using machine learning approaches to gain valuable insights into the complex multimorbidity profiles of patients with cancer. Further studies using similar methodologies may provide deeper insights into the relationships between multimorbidity, mortality, and cancer-specific outcomes, ultimately enabling the incorporation of multimorbidity considerations to improve strategies for the personalized care of patients with cancer.

## Supplementary material

10.2196/71937 Multimedia Appendix 1 Additional file.

## Abbreviations

aHR: adjusted hazard ratio
CVD: cardiovascular disease
HADCL: Hospital Authority Data Collaboration Laboratory
HEI: Healthy Eating Index
*ICD-10*: *International Statistical Classification of Diseases, 10th Revision*
MICE: Multivariate Imputation by Chained Equations
NHANES: National Health and Nutrition Examination Survey
