# Supplementary material for: Association Between Comorbidity Clusters and Mortality in Patients With Cancer: Predictive Modeling Using Machine Learning Approaches of Data From the United States and Hong Kong
Source: JMIR Cancer. 2025 Jul 16;11:e71937. doi: 10.2196/71937 (PMC12286590; doi:10.2196/71937)
Supplement: Multimedia Appendix 1 [file cancer-v11-e71937-s001.docx]

**Table S1.** Guidelines for Developing and Reporting Machine Learning Predictive Models in Biomedical Research.

| Item number | Section | Topic | Checklist item | Page |
| --- | --- | --- | --- | --- |
| 1 | Title | Nature of study | Identify the report as introducing a predictive model | 1 |
| 2 | Abstract | Structured summary | Background  Objectives  Data sources  Performance metrics of the predictive model or models | 2-3 |
| 3 | Introduction | Rationale | Identify the clinical goal  Review the current practice and prediction accuracy of any existing models | 4-5 |
| 4 | Introduction | Objectives | State the nature of study being predictive modeling, defining the target of prediction Identify how the prediction problem may benefit the clinical goal | 5-6 |
| 5 | Methods | Describe the setting | Identify the clinical setting for the target predictive model. Identify the modeling context in terms of facility type, size, volume, and duration of available data | 5-6, 9-10 |
| 6 |  | Define the prediction problem | Define a measurement for the prediction goal. Determine that the study is retrospective or prospective. Identify the problem to be prognostic or diagnostic.  Determine the form of the prediction model: (1) classification if the target variable is categorical, (2) regression if the target variable is continuous, (3) survival prediction if the target variable is the time to an event.  Translate survival prediction into a regression problem, with the target measured over a temporal window following the time of prediction.  Explain practical costs of prediction errors  Defining quality metrics for prediction models.  Define the success criteria for prediction | 8-9,10-11 |
| 7 |  | Prepare data for model building | Identify relevant data sources and quote the ethics approval number for data access.  State the inclusion and exclusion criteria for data.  Describe the time span of data and the sample or cohort size.  Define the observational units on which the response variable and predictor variables are defined.  Define the predictor variables.  Describe the data preprocessing performed, including data cleaning and transformation. Remove outliers with impossible or extreme responses; state any criteria used for outlier removal.  State how missing values were handled.  Describe the basic statistics of the dataset, particularly of the response variable.  Define the model validation strategies. Internal validation is the minimum requirement; external validation should also be performed whenever possible.  Specify the internal validation strategy.  Define the validation metrics. | 6,8-11 |
| 8 |  | Build the predictive model | Identify independent variables that predominantly take a single value. Identify and remove redundant independent variables. Identify the independent variables that may suffer from the perfect separation problem.  Report the number of independent variables, the number of positive examples, and the number of negative examples.  Assess whether sufficient data are available for a good fit of the model.  Determine a set of candidate modeling techniques (eg, logistic regression, random forest, or deep learning).  Define the performance metrics to select the best model.  Specify the model selection strategy. Common methods include K-fold validation or bootstrap to estimate the lost function on a grid of candidate parameter values. | 7-8 |
| 9 | Results | Report the final model and performance | Report the predictive performance of the final model in terms of the validation metrics specified in the methods section.  Comparison with other models in the literature should be based on confidence intervals.  Interpretation of the final model. If possible, report what variables were shown to be predictive of the response variable. State which subpopulation has the best prediction and which subpopulation is most difficult to predict. | 11-20, Supplementary Data |
| 10 | Discussion | Clinical implications | Report the clinical implications derived from the obtained predictive performance. | 22-25 |
| 11 |  | Limitations of the model | Discuss the following potential limitations:  • Assumed input and output data format  • Potential pitfalls in interpreting the model  • Potential bias of the data used in modeling  • Generalizability of the data | 25-26 |
| 12 |  | Unexpected results during the experiments | Report unexpected signs of coefficients, indicating collinearity or complex interaction between predictor variables. | NA |

**Table S2.** Description or categorization of selected covariates for analysis.

| **Variables** | **Categories** |
| --- | --- |
| NHANES |  |
| Ethnicity | Mexican American, Non-Hispanic White, Non-Hispanic Black, or other |
| Family income to poverty ratio | ≤1.3, 1.3–3.5, or >3.5 |
| Educational level | Lower than college, college (or equivalent) or above |
| Cancer type | The types of cancer were categorized according to the body system with reference to the classification provided by the National Cancer Institute [1] in the descriptive analysis and according to their prognosis based on US statistics in the mortality analysis [2,3]: Group 1 (Highest: 5-year survival rate ≥90%); Group 2 (Middle: 5-year survival rate ≥60%); Group 3 (Lowest: 5-year survival rate<60%) |
| Smoking status | Never smokers, former smokers, or current smokers |
| Alcohol consumption | Nondrinkers, low-to-moderate drinkers: <14 drinks/week for men and <7 drinks/week for women, or heavy drinkers: ≥14 drinks/week for men and ≥7 drinks/week for women [4] |
| Physical activity | Inactive, physically active: engaging in moderate or vigorous physical activity for at least l50 minutes per week, irregularly active: less than l50 minutes per week [5] |
| Diet | Regarding diet, the NHANES survey conducted a detailed dietary recall interview to capture participants’ dietary intake. The Healthy Eating Index (HEI), a validated measure of diet quality, was calculated using the dietary recall data to evaluate conformance with federal dietary guidelines in the US. A higher HEI score, with a maximum value of 100, indicates better diet quality [6]. The cut-off for subgroup analysis referenced from the recommended cut-off of the HEI [7]. |
| Supplement use | Participants were asked about their supplement use in the past 30 days. Those who reported using any supplements were further asked to provide information about the duration of use. In this study, we considered supplement users as individuals who had taken any supplements (including vitamins, minerals, botanicals, amino acids, or others) for more than 90 days. |
| Body Mass Index (BMI) | Normal weight (<25 kg/m2), overweight (25–30 kg/m2), or obese (≥30 kg/m2). |
| HADCL |  |
| Income level | The residential areas (district) were categorized into 3 groups based on the latest median monthly household income by districts published in 2023 [8]: lowest-income, middle-income, highest-income. |
| Cancer type | The types of cancer were grouped by ICD-10 diagnosis code in Hong Kong and according to their prognosis based on local and US statistics in the mortality analysis [2,3,9]. |

**References:**

1. Centers for Disease Control and Prevention. United States Cancer Statistics: Survival. <https://gis.cdc.gov/Cancer/USCS/#/Survival/> (accessed Jul 2 2024)

2. National Cancer Institute Surveillance, Epidemiology and End Results Program. Cancer Stat Facts: Common Cancer Sites. <https://seer.cancer.gov/statfacts/html/common.html> (accessed Jul 2 2024)

3. National Cancer Institute. Cancers by Body Location/System 2023. <https://www.cancer.gov/types/by-body-location>. (accessed Jul 2 2024)

4. DeSalvo KB, Olson R, Casavale KO. Dietary guidelines for Americans. *Jama* 2016; **315**(5): 457-8.

5. Piercy KL, Troiano RP, Ballard RM, Carlson SA, Fulton JE, Galuska DA, et al. The physical activity guidelines for Americans. *Jama* 2018; **320**(19): 2020-8.

6. Guenther PM, Kirkpatrick SI, Reedy J, Krebs-Smith SM, Buckman DW, Dodd KW, et al. The Healthy Eating Index-2010 is a valid and reliable measure of diet quality according to the 2010 Dietary Guidelines for Americans. *The Journal of nutrition* 2014; **144**(3): 399-407

7. Krebs-Smith SM, Pannucci TE, Subar AF, Kirkpatrick SI, Lerman JL., Tooze JA, et al. Update of the healthy eating index: HEI-2015. *Journal of the Academy of Nutrition and Dietetics* 2018; **118**(9): 1591-1602.

8. Census and Statistics Department, HKSAR. Population and Household Statistics Analysed by District Council District
 <https://www.censtatd.gov.hk/wbr/B1130301/B11303012023AN23/att/en/B11303012023AN23.pdf> (accessed Jul 2 2024)

9. Hospital Authority. Hong Kong Cancer Registry. <https://www3.ha.org.hk/cancereg/default.asp> (accessed Jul 2 2024)

**Table S3.** Performance metrics of the four clustering approaches.

| Parameters | Bernoulli mixture models | K-modes | K-medoids | Bisecting K-medoids |
| --- | --- | --- | --- | --- |
| Silhouette score | 0.168 | 0.157 | 0.146 | 0.165 |
| CH index | 411.11 | 402.03 | 398.88 | 410.78 |
| DB index | 2.37 | 2.53 | 2.50 | 2.42 |

^a^ Different performance measures have been used, including Silhouette scores, Calinski–Harabasz index, and Davies-Bouldin Index. A high Silhouette score, high CH index and low DB index indicate better performance of clusters.

**Table S4.** Baseline characteristics of included participants by comorbidity clusters in the NHANES cohort.

|  | Total | Cluster 1 (Low Comorbidity) | Cluster 2 (Metabolic) | Cluster 3 (CVD) | Cluster 4 (Respiratory) |
| --- | --- | --- | --- | --- | --- |
|  | N=4,390 (%) | N=2,127 (%) | N=1,525 (%) | N=421 (%) | N=317 (%) |
| Socio-demographics |  |  |  |  |  |
| Sex  Male  Female | 2,014 (45.9)  2,376 (54.2) | 893 (42.0)  1,234 (58.0) | 747 (49.0)  778 (51.0) | 270 (64.1)  151 (35.9) | 104 (32.8)  213 (67.2) |
| Age (Mean [SD]) | 66.0 [14.6] | 61.6 [16.5] | 70.5 [10.3] | 73.1 [9.7] | 64.5 [13.8] |
| Education level  Below College  College or above | 2,154 (49.1)  2,236 (50.9) | 975 (45.8)  1,152 (54.2) | 781 (51.2)  744 (48.8) | 239 (56.8)  182 (43.2) | 158 (50.2)  159 (49.8) |
| Family income to poverty (Mean [SD])  ≤1.3  1.3-3.5  >3.5 | 2.65 [1.59]  1,184 (27.0)  1,803 (41.0)  1,404 (32.0) | 2.86 [1.64]  517 (24.3)  807 (37.9)  803 (37.8) | 2.57 [1.53]  413 (27.1)  675 (44.3)  437 (28.6) | 2.37 [1.49]  128 (30.4)  191 (45.4)  102 (24.2) | 2.09 [1.40]  125 (39.4)  130 (41.0)  62 (19.6) |
| Ethnicities  Mexican American  Non-Hispanic White  Non-Hispanic Black  Others | 344 (7.8)  2,870 (65.4)  703 (16.0)  473 (10.8) | 198 (9.3)  1,398 (65.7)  286 (13.5)  245 (11.5) | 113 (7.4)  934 (61.2)  317 (20.8)  161 (10.6) | 19 (4.5)  320 (76.0)  53 (12.6)  29 (6.9) | 14 (4.4)  218 (68.8)  47 (14.8)  38 (12.0) |
| Clinical |  |  |  |  |  |
| Age at cancer diagnosis (Mean [SD]) | 55.4 [17.6] | 51.3 [18.7] | 59.7 [14.9] | 62.9 [14.8] | 53.0 [17.2] |
| Time since cancer diagnosis (Median no. of years [IQR]) | 7 [3-15] | 7 [3-14] | 7 [3-15] | 7 [2-15] | 9 [3-18] |
| Type of cancer  Breast cancer  Digestive/Gastrointestinal cancer  Genitourinary cancer  Gynecological cancer  Skin cancer  Head and neck cancer  Respiratory/Thoracic cancer  Others | 815 (18.6)  536 (12.2)  1,102 (25.1)  694 (15.8)  762 (17.4)  173 (3.9)  152 (3.5)  552 (12.6) | 408 (19.2)  220 (10.3)  447 (21.0)  383 (18.0)  350 (16.5)  105 (4.9)  53 (2.5)  319 (15.0) | 299 (19.6)  204 (13.4)  459 (30.1)  205 (13.4)  277 (18.2)  40 (2.6)  48 (3.1)  145 (9.5) | 48 (11.4)  68 (16.2)  149 (35.4)  37 (8.8)  77 (18.3)  12 (2.9)  21 (5.0)  56 (13.3) | 60 (18.9)  44 (13.9)  47 (14.8)  69 (21.8)  58 (18.3)  16 (5.0)  30 (9.5)  32 (10.1) |
| Cancer prognosis  Highest  Middle  Lowest | 2512 (57.2)  1435 (32.7)  443 (10.1) | 1209 (56.8)  729 (34.3)  189 (8.9) | 915 (60.0)  457 (30.0)  153 (10.0) | 239 (56.8)  138 (32.8)  44 (10.5) | 149 (47.0)  111 (35.0)  57 (18.0) |
| Comorbidities  Hyperlipidemia  Hypertension  Arthritis  Heart failure Coronary heart diseases  Angina  Heart attack  Stroke  Bronchitis  Liver condition  Kidney diseases  Diabetes  Asthma  Thyroid diseases  Emphysema | 2,234 (50.9)  2,812 (64.1)  2,206 (50.3)  347 (7.9)  426 (9.7)  289 (6.6)  443 (10.1)  404 (9.2)  452 (10.3)  255 (5.8)  338 (7.7)  1,074 (24.5)  678 (15.4)  320 (7.3)  263 (6.0) | 715 (33.6)  850 (40.0)  649 (30.5)  9 (0.4)  10 (0.5)  8 (0.4)  15 (0.7)  21 (1.0)  89 (4.2)  67 (3.1)  35 (1.6)  102 (4.8)  211 (9.9)  7 (0.3)  31 (1.5) | 1,075 (70.5)  1,412 (92.6)  1,055 (69.2)  87 (5.7)  91 (6.0)  61 (4.0)  89 (5.8)  239 (15.7)  39 (2.6)  96 (6.3)  181 (11.9)  697 (45.7)  157 (10.3)  226 (14.8)  40 (2.6) | 278 (66.0)  351 (83.4)  262 (62.2)  211 (50.1)  320 (76.0)  192 (45.7)  309 (73.4)  98 (23.3)  66 (15.7)  34 (8.1)  72 (17.1)  164 (39.0)  84 (20.0)  50 (11.9)  65 (15.7) | 166 (52.4)  199 (62.8)  240 (75.7)  40 (12.6)  5 (1.6)  28 (8.8)  30 (9.5)  46 (14.5)  258 (81.4)  58 (18.3)  50 (15.8)  111 (35.0)  226 (71.3)  37 (11.7)  127 (40.1) |
| Lifestyle |  |  |  |  |  |
| Body Mass Index (Mean [SD])  <25 kg/m^2^ (normal)  25-30 kg/m^2^ (overweight)  ≥30 kg/m^2^ (obese) | 28.9 [6.6]  1,266 (28.8)  1,549 (35.3)  1,575 (35.9) | 27.8 [6.0]  755 (35.5)  730 (34.3)  642 (30.2) | 30.0 [6.6]  325 (21.3)  579 (38.0)  621 (40.7) | 29.1 [6.0]  106 (25.2)  145 (34.4)  170 (40.3) | 30.4 [8.5]  79 (24.9)  96 (30.3) 143 (44.8) |
| Smoking status  Never smokers  Former smokers  Current smokers | 1,923 (43.8)  1,766 (40.2)  701 (16.0) | 1,017 (47.8)  736 (34.6)  374 (17.6) | 680 (44.6)  677 (44.4)  168 (11.0) | 138 (32.8)  217 (51.5)  66 (15.7) | 88 (27.8)  136 (42.9)  93 (29.3) |
| Drinking status  Non-drinker  Low-to-moderate drinker  Heavy drinker | 2,202 (50.2)  1,885 (42.9)  303 (6.9) | 964 (45.3)  994 (46.7)  169 (8.0) | 821 (53.8)  613 (40.2)  91 (6.0) | 247 (58.7)  154 (36.6)  20 (4.7) | 170 (53.6)  124 (39.1)  23 (7.3) |
| Physical activity  Physically active (≥150 hours/week)  Irregularly active (<150 hours/week)  Inactive | 1,158 (26.4)  680 (15.5)  2,552 (58.1) | 696 (32.7)  352 (16.6)  1,079 (50.7) | 337 (22.1)  233 (15.3)  955 (62.6) | 71 (16.9)  49 (11.6)  301 (71.7) | 54 (17.0)  46 (14.5)  217 (68.5) |
| Healthy eating index (Median [IQR])  <51.55  ≥51.55 | 52 [42-63]  2194 (50.0)  2196 (50.0) | 51 [41-62]  1106 (52.0)  1021 (48.0) | 53 [42-63]  708 (46.4)  817 (53.6) | 52 [43-63]  209 (49.6)  212 (50.4) | 50 [41-59]  171 (53.9)  146 (46.1) |
| Supplement use (≥90 days) | 2,646 (60.3) | 1,262 (59.3) | 943 (61.8) | 256 (60.8) | 185 (58.4) |

^a^ Cancer prognosis was based on the US statistics (: Group 1 (Highest: average 5-year survival rate ≥90%); Group 2 (Middle: average 5-year survival rate ≥60% and <90%); and Group 3 (Lowest: average 5-year survival rate <60%). [Reference: Centers for Disease Control and Prevention. United States Cancer Statistics: Survival. https://gis.cdc.gov/Cancer/USCS/#/Survival/ (accessed Jul 2 2024); National Cancer Institute Surveillance, Epidemiology and End Results Program. Cancer Stat Facts: Common Cancer Sites. https://seer.cancer.gov/statfacts/html/common.html (accessed Jul 2 2024)]

**Table S5.** Distribution of comorbidities among four comorbidity clusters using Bernoulli Mixture Models in the NHANES cohort.

| Cluster | Total | **Cardiovascular diseases (CVD)** | | | | | **Metabolic syndromes** | | | **Respiratory diseases** | | | **Others** | | | | **Characteristics** |
| --- | --- | --- | --- | --- | --- | --- | --- | --- | --- | --- | --- | --- | --- | --- | --- | --- | --- |
|  |  | Heart failure | Coronary heart disease | Angina | Heart attack | Stroke | Hyper-tension | Hyper-lipidemia | Diabetes | Bronchitis | Emphy-sema | Asthma | Liver | Thyroid | Kidney | Arthritis |  |
| 1st | **2127** | 0.42 | 0.47 | 0.38 | 0.71 | 0.99 | 39.96 | 33.62 | 4.80 | 4.18 | 1.46 | 9.92 | 3.15 | 0.33 | 1.65 | 30.51 | Low level of comorbidities |
| 2nd | **1525** | 5.70 | 5.97 | 4.00 | 5.84 | 15.67 | 92.59 | 70.49 | 45.70 | 2.56 | 2.62 | 10.30 | 6.30 | 14.82 | 11.87 | 69.18 | Highest metabolic diseases (+ High Arthritis) |
| 3rd | **421** | 50.12 | 76.01 | 45.61 | 73.40 | 23.28 | 83.37 | 66.03 | 38.95 | 15.68 | 15.44 | 19.95 | 8.08 | 11.88 | 17.10 | 62.23 | Highest CVD + High metabolic diseases (2^nd^) (+ High Arthritis) |
| 4th | **317** | 12.62 | 1.58 | 8.83 | 9.46 | 14.51 | 62.78 | 52.37 | 35.02 | 81.39 | 40.06 | 71.29 | 18.30 | 11.67 | 15.77 | 75.71 | High respiratory diseases + Moderate metabolic diseases (3^rd^) (+ High Arthritis) |

**Table S6.** Baseline characteristics of included participants by comorbidity clusters in the HADCL cohort.

|  | Total | Cluster 1 (Low Comorbidity) | Cluster 2 (Metabolic) | Cluster 3 (CVD) | Cluster 4 (Respiratory) |
| --- | --- | --- | --- | --- | --- |
|  | N=12,484 (%) | N=7.392 (%) | N=2,521 (%) | N=2,188 (%) | N=383 (%) |
| Socio-demographics |  |  |  |  |  |
| Sex  Male  Female | 5,900 (47.3)  6,584 (52.7) | 3,169 (42.9)  4,223 (57.1) | 1,230 (48.8)  1,291 (51.2) | 1,307 (59.7)  881 (40.3) | 194 (50.7)  189 (49.3) |
| Age (Mean [SD]) ^a^ | 60.9 [14.4] | 56.0 [13.8] | 65.9 [12.0] | 70.5 [11.2] | 69.2 [12.9] |
| Income level ^b^  Lowest-income  Middle-income  Highest-income | 5,332 (42.7)  3,642 (29.2)  3,510 (28.1) | 3,172 (42.9)  2,200 (29.8)  2,020 (27.3) | 1,057 (41.9)  711 (28.2)  753 (29.9) | 940 (43.0)  624 (28.5)  624 (28.5) | 163 (42.6)  107 (27.9)  113 (29.5) |
| Clinical |  |  |  |  |  |
| Age at cancer diagnosis (Mean [SD]) | 64.8 [14.7] | 59.9 [14.2] | 70.3 [12.6] | 73.7 [11.7] | 73.1 [13.0] |
| Type of cancer  Cancers of lip, oral cavity and pharynx (C00-C14)  Cancers of digestive organs (C15-C26)  Cancers of respiratory and intrathoracic organs (C30-C39)  Cancers of bone and articular cartilage (C40-C41)  Malignant melanoma of skin (C43)  Cancers of mesothelial and soft tissue (C45-C49)  Breast cancer (C50)  Cancers of female genital organs (C51-C58)  Cancers of male genital organs (C60-C63)  Cancers of urinary tract (C64-C68)  Cancer of eye, brain and other parts of CNS (C69-C72)  Cancers of thyroid and other endocrine glands (C73-C75)  Cancers of ill-defined, secondary and unspecified sites (C76-C80)  Cancers of primary, of lymphoid, hematopoietic and related tissue (C81-C96) | 863 (6.9)  3,938 (31.5)  1,741 (13.9)  50 (0.4)  49 (0.4)  162 (1.3)  2,242 (18.0)  998 (8.0)  898 (7.2)  772 (6.2)  96 (0.8)  430 (3.4)  3,172 (25.4)  810 (6.5) | 589 (8.0)  2,037 (27.6)  946 (12.8)  34 (0.5)  25 (0.3)  106 (1.4)  1,644 (22.2)  671 (9.1)  437 (5.9)  334 (4.5)  61 (0.8)  322 (4.4)  1,807 (24.4)  493 (6.7) | 135 (5.4)  964 (38.2)  356 (14.1)  7 (0.3)  12 (0.5)  ‘30 (1.2)  356 (14.1)  180 (7.1)  207 (8.2)  176 (7.0)  17 (0.7)  62 (2.5)  706 (28.0)  146 (5.8) | 121 (5.5)  815 (37.2)  358 (16.4)  7 (0.3)  9 (0.4)  23 (1.1)  198 (9.1)  125 (5.7)  214 (9.8)  228 (10.4)  17 (0.8)  42 (1.9)  556 (25.4)  149 (6.8) | 18 (4.7)  122 (31.9)  81 (21.1)  2 (0.5)  3 (0.8)  3 (0.8)  44 (11.5)  22 (5.7)  40 (10.4)  34 (8.9)  1 (0.3)  4 (1.1)  103 (28.9)  22 (5.7) |
| Cancer prognosis ^c^  Highest  Middle  Lowest | 2,819 (22.6)  3,839 (30.7)  5.826 (46.7) | 1,967 (26.6)  2,331 (31.5)  3,094 (41.9) | 453 (18.0)  726 (28.8)  1,342 (53.2) | 333 (15.2)  669 (30.6)  1,186 (54.2) | 66 (17.2)  113 (29.5)  204 (53.3) |
| Comorbidities  Hyperlipidemia  Hypertension  Arthritis  Heart failure Coronary heart diseases  Angina  Heart attack  Stroke  Bronchitis  Liver condition  Kidney diseases  Diabetes  Asthma  Thyroid diseases  Emphysema | 1,136 (9.1)  3,464 (27.7)  513 (4.1)  801 (6.4)  1,047 (8.4)  397 (3.2)  484 (3.9)  905 (7.2)  137 (1.1)  1,265 (10.1)  712 (5.7)  2,053 (16.4)  246 (2.0)  623 (5.0)  14 (0.1) | 0 (0)  0 (0)  174 (2.4)  0 (0)  0 (0)  0 (0)  0 (0)  0 (0)  0 (0)  585 (7.9)  122 (1.7)  0 (0)  0 (0)  361 (4.9)  0 (0) | 509 (20.2)  1,971 (78.2)  167 (6.6)  0 (0)  0 (0)  0 (0)  0 (0)  0 (0)  0 (0)  339 (13.5)  245 (9.7)  1,174 (46.6)  0 (0)  128 (5.1)  0 (0) | 578 (26.4)  1,298 (59.3)  140 (6.4)  710 (32.5)  978 (44.7)  367 (16.8)  447 (20.4)  853 (39.0)  0 (0)  276 (12.6)  314 (14.4)  783 (35.8)  0 (0)  117 (5.4)  0 (0) | 49 (12.8)  195 (50.9)  32 (8.4)  91 (23.8)  69 (18.0)  30 (7.8)  37 (9.7)  52 (13.6)  137 (35.8)  65 (17.0)  31 (8.1)  96 (25.1)  246 (64.2)  17 (4.4)  14 (3.7) |

^a^This refers to the age in 2007 (the first time-point for patient sampling).

^b^The income level is based on the residential areas of individuals (which were categorized into 3 groups based on median monthly household income)

^c^Cancer diagnoses were further classified according to their prognosis considering our previous classification in the NHANES cohort and local statistics.

**Table S7.** Distribution of comorbidities among four comorbidity clusters in the HADCL cohort.

| Cluster | Total | **Cardiovascular diseases (CVD)** | | | | | **Metabolic syndromes** | | | **Respiratory diseases** | | | **Others** | | | | **Characteristics** |
| --- | --- | --- | --- | --- | --- | --- | --- | --- | --- | --- | --- | --- | --- | --- | --- | --- | --- |
|  |  | Heart failure | Coronary heart disease | Angina | Heart attack | Stroke | Hyper-tension | Hyper-lipidemia | Diabetes | Bronchitis | Emphy-sema | Asthma | Liver | Thyroid | Kidney | Arthritis |  |
| 1st | 7392 | 0.00 | 0.00 | 0.00 | 0.00 | 0.00 | 0.00 | 0.00 | 0.00 | 0.00 | 0.00 | 0.00 | 7.91 | 4.88 | 1.65 | 2.35 | Low level of comorbidities |
| 2nd | 2521 | 0.00 | 0.00 | 0.00 | 0.00 | 0.00 | 78.18 | 20.19 | 46.57 | 0.00 | 0.00 | 0.00 | 13.45 | 5.08 | 9.72 | 6.62 | High metabolic diseases |
| 3rd | 2188 | 32.45 | 44.70 | 16.77 | 20.43 | 38.99 | 59.32 | 26.42 | 35.79 | 0.00 | 0.00 | 0.00 | 12.61 | 5.35 | 14.35 | 6.40 | High CVD + High metabolic diseases (2^nd^) |
| 4th | 383 | 23.76 | 18.02 | 7.83 | 9.66 | 13.58 | 50.91 | 12.79 | 25.07 | 35.77 | 3.66 | 64.23 | 16.97 | 4.44 | 8.09 | 8.36 | High respiratory diseases + moderate metabolic diseases (3^rd^) |

**Figure S1.** Visualization of the distribution of comorbidities across clusters using four different machine learning methods.

|  |  |
| --- | --- |
| Bernoulli mixture model | Bisecting K-medoids |
| Median rank: 1 | Median rank: 2 |
|  |  |
| K-medoids | K-modes |
| Median rank: 3 | Median rank: 3.75 |

**Figure S2.** Distribution of comorbidities among the four comorbidity clusters in the HADCL cohort.

^a^Cluster 1 (Low Comorbidity Cluster): characterized by significantly lower percentages of all comorbidities than the patients in the other clusters; Cluster 2 (Metabolic Cluster): characterized by the highest burden of metabolic syndrome; Cluster 3 (CVD Cluster): characterized by the highest burden of cardiovascular diseases among the clusters, with a relatively high burden of metabolic syndromes (although lower than that of Cluster 2); Cluster 4 (Respiratory Cluster): characterized by a significantly higher burden of respiratory diseases, with a moderate burden of metabolic syndromes (although lower than those of Clusters 2 and 3).


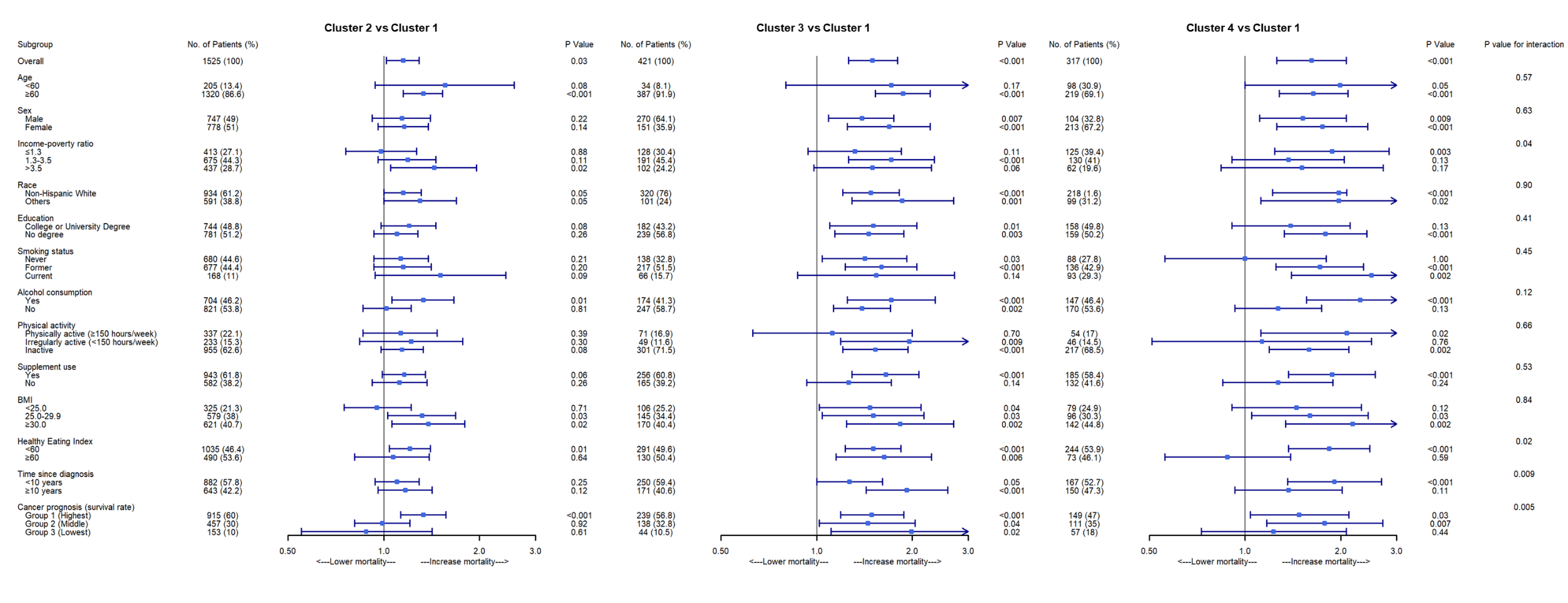
**Figure S3.** Effect modification by factors on the association of clusters with mortality in the NHANES cohort.
